# Enolase AmEno15, a Promising Candidate for Understanding the Infectious Process of *Anaplasma marginale*

**DOI:** 10.3390/ijms26189093

**Published:** 2025-09-18

**Authors:** Maria del Socorro López-López, Hugo Aguilar-Díaz, Armando Burgos-Solorio, Rosa Estela Quiroz-Castañeda

**Affiliations:** 1 Centro de Investigaciones Biológicas, Universidad Autónoma del Estado de Morelos, Cuernavaca 62209, Morelos, Mexico; maria.lopez1@uaem.edu.mx (M.d.S.L.-L.); burgos@uaem.mx (A.B.-S.); 2 Centro Nacional de Investigaciones Disciplinarias en Salud Animal e Inocuidad, Instituto Nacional de Investigaciones Forestales, Agrícolas y Pecuarias (CENID-SAI, INIFAP), Jiutepec 62574, Morelos, Mexico; aguilar.hugo@inifap.gob.mx

**Keywords:** red blood cells, moonlighting proteins, cattle, pathogens, tick gut

## Abstract

Bovine anaplasmosis is a serious health problem in the livestock industry. Currently, there is a lack of information regarding the molecular mechanisms by which *Anaplasma marginale* adheres and invades bovine erythrocytes. Even more is unknown about how it binds to tick gut cells when the tick feeds on infected blood. In other pathogens, enolase has been shown to play a significant role in adhesion to host tissue, serving as the first step in invasion and colonization. Therefore, the elucidation of the role of the moonlighting protein enolase AmEno15 of *A. marginale* in the adhesion to erythrocytes, tick gut tissue, and plasminogen is addressed in this work. We explored the role of *A. marginale* recombinant AmEno15 in the adhesion to spectrin, stomatin, fibronectin, and plasminogen. Firstly, we modeled AmEno15 tridimensionally and performed a molecular dynamics approach to determine whether AmEno15 could bind to the proteins mentioned above. Then, we expressed recombinant AmEno15 and performed a microplate binding assay using fixed concentrations of the erythrocyte proteins, fibronectin, and plasminogen, as well as variable concentrations of AmEno15. We found that AmEno15 binds to all assessed proteins in a specific and concentration-dependent manner. Spectrin and fibronectin-AmEno15 binding occurs at high concentrations, while stomatin and plasminogen-AmEno15 binding occurs at lower concentrations. Our findings bring us closer to understanding the role of the moonlighting protein enolase and suggest its participation in the *A. marginale* adhesion and invasion processes, providing a basis for the control of tick-borne diseases.

## 1. Introduction

The pathogen *Anaplasma marginale,* the causative agent of bovine anaplasmosis, is a Gram-negative bacterium that infects red blood cells. In cattle, anaplasmosis is characterized by anemia, jaundice, abortions, loss of milk and meat production, and even death [1,2]. This disease is primarily transmitted by ticks of the genera *Rhipicephalus* and *Dermacentor*, which act as biological vectors, as well as by veterinary surgical materials and blood-sucking flies, considered mechanical vectors [3]. Bovine anaplasmosis’s most significant impact is on the production of dairy and meat derivatives. Therefore, addressing economic losses is a priority not only in the world’s tropical regions where vectors are abundant but also in cold regions to which ticks have migrated due to climate change [4,5]. To date, efforts to develop an effective vaccine for bovine anaplasmosis have been unsuccessful. Thus, the search for new candidates demands powerful and novel bioinformatics strategies in vaccine design based on proteins different from those typically analyzed (Major Surface Proteins (MSPs), Outer Membrane Proteins (OMPs), Type 4 Secretion System (T4SS) proteins) [6,7]. In this regard, recent genomic approaches to *A. marginale* Mexican strains elucidated moonlighting proteins (MLPs) as potential vaccine candidates against anaplasmosis. They play roles in invasion, adhesion, and virulence, as reported in other pathogens [8,9,10]. The functional versatility of MLPs represents an advantage for many pathogens, enabling them to perform multiple functions despite having a reduced genome, as seen in *A. marginale* [11,12]. The primary characteristic of MLPs is their ability to perform a canonical function in primary metabolism and a distinct secondary function in other cellular locations [13]. In this regard, enolase plays a primary role in glycolysis and has also been proposed as a virulence factor due to its ability to initiate tissue invasion through adhesion to host cells, making it a considered immunogenic target against bacteria and parasites [14,15,16,17]. Recently, the identification of enolase AmEno15 in *A. marginale* has been reported; however, its role in anaplasmosis pathogenesis has not been elucidated [18]. Enolase functions in other pathogenic bacteria are better understood. For instance, in *Mycoplasma bovis* and *Mycoplasma hyopneumoniae*, the enolase binds to plasminogen and activates it in plasmin, which facilitates the bacterial invasion into embryonic bovine lung and swine tracheal cells, respectively [19,20]. It is known that *Mycoplasma suis* enolase adheres to porcine red blood cells, and, in particular, the recombinant enolase induces an immune response that confers partial resistance to mycoplasmosis in piglets [21,22]. Additionally, some reports highlight the ability of enolase to bind to extracellular matrix proteins, including fibronectin [9], vitronectin [23], laminin [24,25], and collagen [26], which supports the role of the enolase as an adhesive protein. Unfortunately, the mechanism by which *A. marginale* adheres to and infects bovine erythrocytes remains unclear. Interestingly, molecular docking studies predict that AmEno15 could bind to erythrocyte membrane proteins and extracellular matrix proteins [27,28]. This information is crucial to understanding how *A. marginale* enters its target cell. In this work, we use a deep immunoinformatics approach to predict AmEno15 binding to erythrocyte membrane proteins (spectrin and stomatin), extracellular matrix proteins from tick gut tissue (fibronectin), and plasminogen. We also experimentally show that AmEno15 effectively binds to these proteins. These results contribute to understanding the role of AmEno15 in mediating pathogen interactions with vector and host, its involvement in adherence and invasion of erythrocytes, and its probable molecular interaction with tick tissues, a key step in pathogen transmission.

## 2. Results

### 2.1. AmEno15 Three Dimensional Modeling

**Figure 1:**
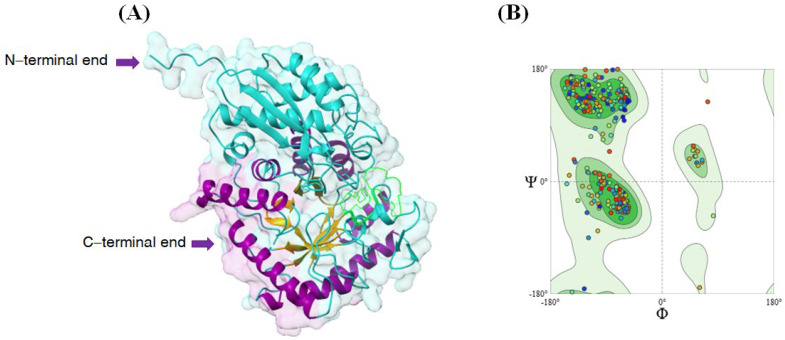
3D model and assessment of AmEno15. (**A**) AmEno15 modeled on *A. marginale* enolase monomer (Q5PAS6.1.A) in AlphaFold v2 SwissModel server. The 3D structure reveals the classical enolase topology of a two-layer sandwich in the N-terminal end (cyan) and an alpha-beta barrel (TIM barrel) in the C-terminal end (purple/golden). (**B**) Ramachandran plot for the AmEno15 3D model showed that most of the amino acids are in the favored regions according to the Phi (Φ) and Psi (Ψ) angle values, suggesting the 3D model is feasible. Each dot on the plot represents the Φ and Ψ angles for one amino acid residue in AmEno15 protein.

A 3D model of AmEno15 monomer was generated using the enolase template from *A. marginale* AlphaFold v2 DB (Q5PAS6.1.A), with an average pLDDT of 95.40%, 100% sequence identity, and 80% coverage. A Ramachandran plot indicated that 95.10% of amino acids resided in favored regions, confirming that the AmEno15 3D model has high resolution, as scores above 90% correspond to protein structures with a resolution of 2–3 Å (Figure 1). The AmEno15 3D model achieved a MolProbity score of 1.24, demonstrating its high quality. This score integrates clashscore (steric clashes), Ramachandran, and rotamer statistics, normalized to the X-ray resolution scale.

### 2.2. AmEno15 Recombinant Expression

**Figure 2:**
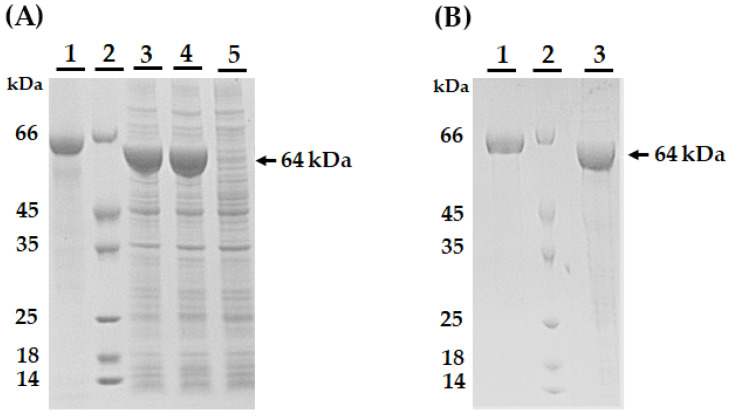
Expression and purification of recombinant AmEno15-Trx-6His. (**A**) SDS-PAGE 12% analysis of AmEno15-Trx-6His induction under different conditions: Line 1, Bovine serum albumin (BSA, 0.4 mg/mL); Line 2, molecular weight marker; Line 3, sample induced with 0.8 mM IPTG for 4 h at 37 °C; Line 4, sample induced with 0.8 mM IPTG for 16 h at 16 °C. Recombinant AmEno15-Trx-6His appears as a 64 kDa band (black arrow). The chosen expression condition was 0.8 mM IPTG for 4 h at 37 °C. (**B**) SDS-PAGE 12% analysis of purified AmEno15-Trx-6His: Line 1, BSA, 0.4 mg/mL; Line 2, molecular weight marker; Line 3, purified AmEno15-Trx-6His dialyzed with PBS (64 kDa, black arrow).

The recombinant expression of AmEno15-Trx-6His resulted in a protein with a molecular weight of 64 kDa (Figure 2), which is consistent with the predicted molecular weight of 64.57 kDa by ProtParam, corresponding to 587 amino acids.

### 2.3. Molecular Docking and Molecular Dynamics Simulation

**Figure 3:**
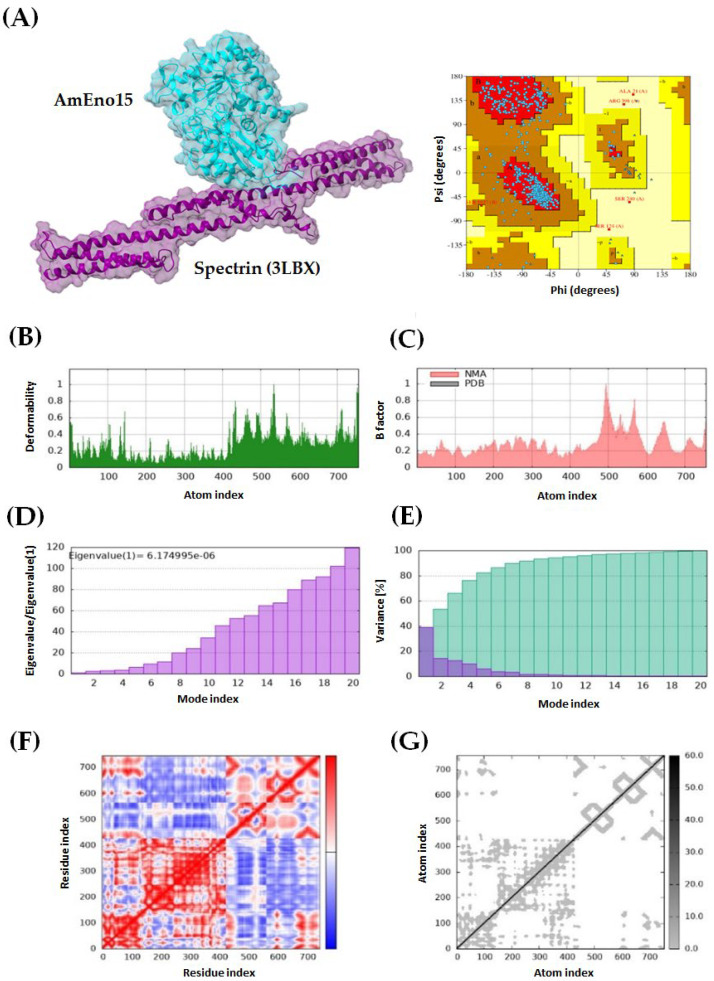
Molecular dynamics simulation of AmEno15-spectrin (3LBX) docking complex. (**A**) 3D model and Ramachandran plot assessment of the AmEno15-spectrin interaction. Each dot on the plot represents the Φ and Ψ angles for each amino acid residue in the docking. For the docking complex molecular dynamics simulation: (**B**) The deformability (green graph) reflects the degree of deformability (0–1 Å); (**C**) The B-factor (pink graph) visualizes flexibility and movement; (**D**) Eigenvalues (purple graph) indicate the energy required for deformation, with lower values showing easier deformation; (**E**) The variance map (cyan bars) identifies complex parts more likely to move during conformational change; (**F**) The covariance map highlights correlated regions between linked amino acids (white: uncorrelated, red: correlated, blue: anti-correlated); and (**G**) The elastic network model displays pairs of atoms connected by springs (each dot represents a spring; light grey dots indicate more flexibility).

**Figure 4:**
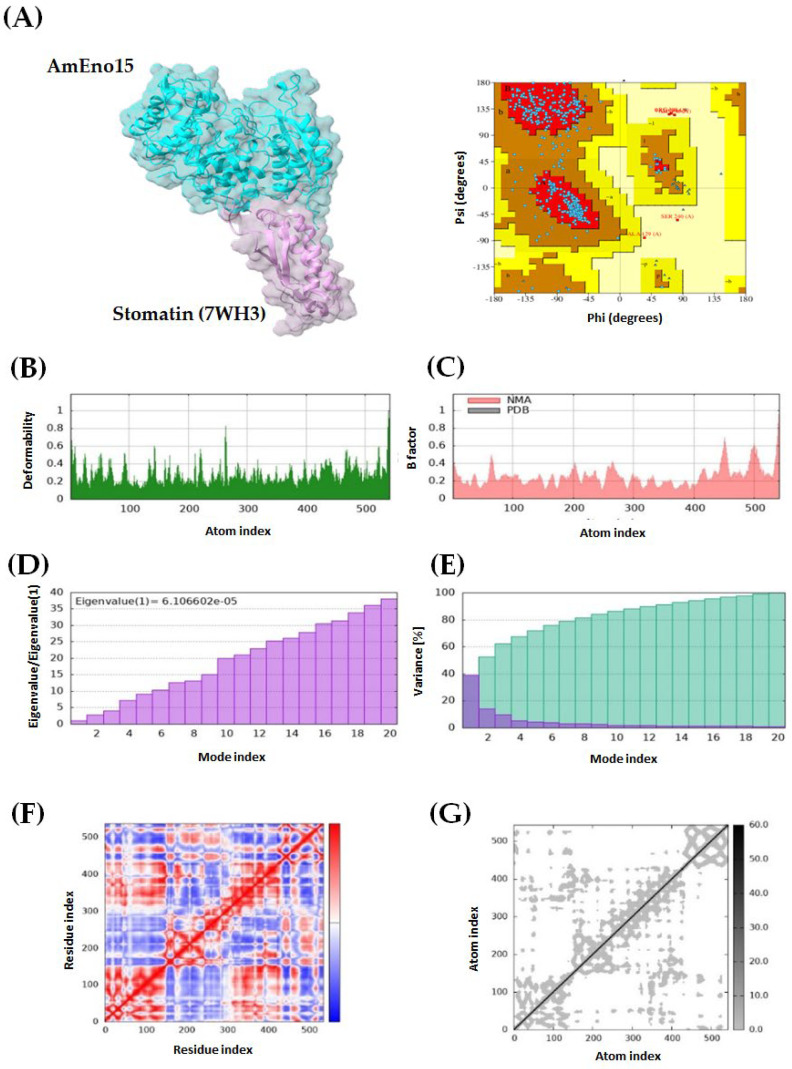
Molecular dynamics simulation of AmEno15-stomatin (7WH3) docking complex. (**A**) A 3D model and Ramachandran plot assessment of the AmEno15-stomatin interaction. Each dot on the plot represents the Φ and Ψ angles for each amino acid residue in the docking. For the docking complex molecular dynamics simulation: (**B**) The deformability (green graph) reflects the degree of deformability (0–1 Å). (**C**) The B-factor (pink graph) visualizes flexibility and movement. (**D**) Eigenvalues (purple graph) indicate the energy required for deformation, with lower values showing easier deformation. (**E**) The variance map (cyan bars) identifies complex parts more likely to move during conformational change. (**F**) The covariance map highlights correlated regions between linked amino acids (white: uncorrelated, red: correlated, blue: anti-correlated). (**G**) The elastic network model displays pairs of atoms connected by springs (each dot represents a spring; light grey dots indicate more flexibility).

**Figure 5:**
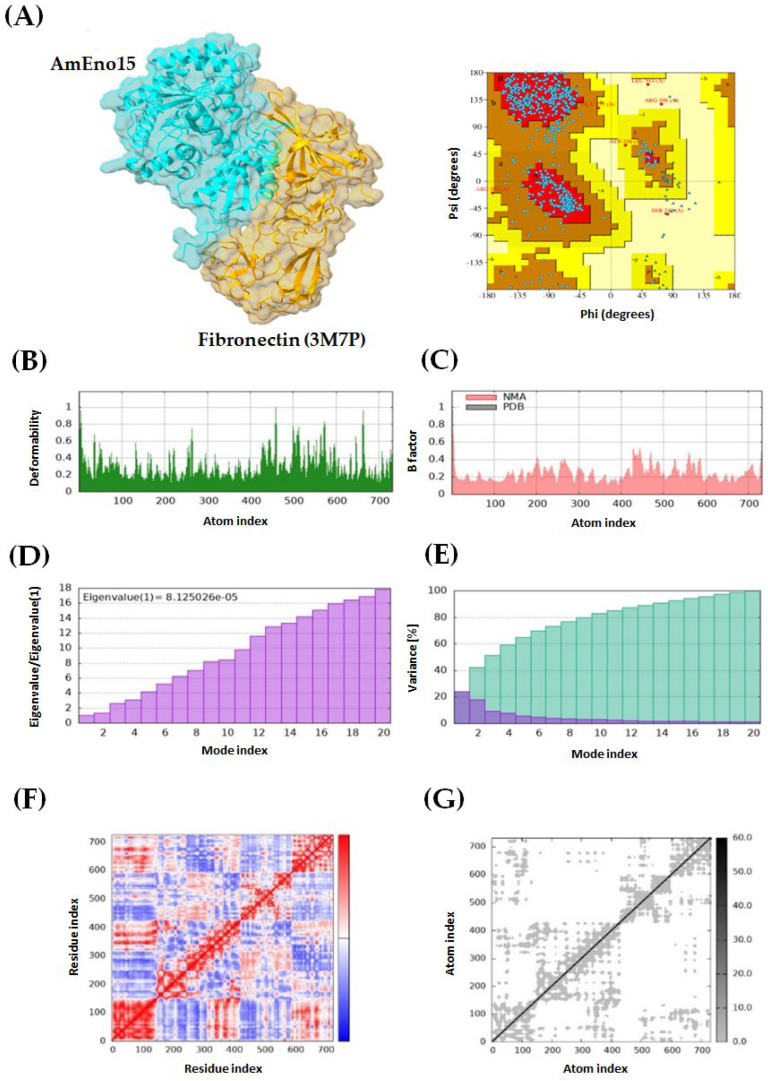
Molecular dynamics simulation of AmEno15-fibronectin (3M7P) docking complex. (**A**) A 3D model and Ramachandran plot assessment of the AmEno15–fibronectin interaction. Each dot on the plot represents the Φ and Ψ angles for each amino acid residue in the docking. For the docking complex molecular dynamics simulation: (**B**) The deformability (green graph) reflects the degree of deformability (0–1 Å). (**C**) The B-factor (pink graph) visualizes flexibility and movement. (**D**) Eigenvalues (purple graph) indicate the energy required for deformation, with lower values showing easier deformation. (**E**) The variance map (cyan bars) identifies complex parts more likely to move during conformational change. (**F**) The covariance map highlights correlated regions between linked amino acids (white: uncorrelated, red: correlated, blue: anti-correlated). (**G**) The elastic network model displays pairs of atoms connected by springs (each dot represents a spring; light grey dots indicate more flexibility).

**Figure 6:**
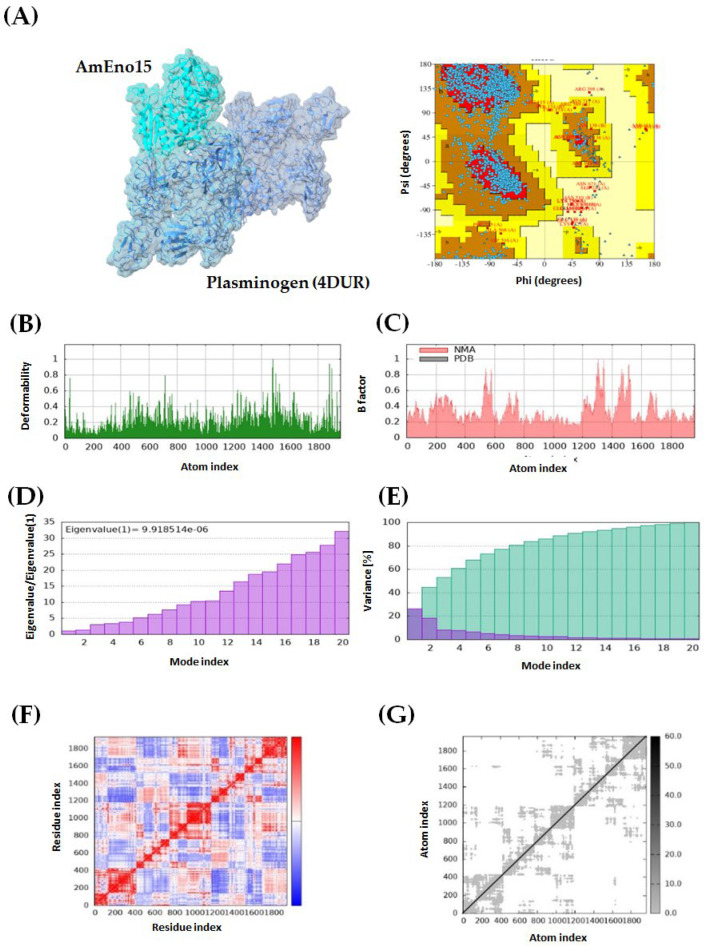
Molecular dynamics simulation of AmEno15-plasminogen (4DUR) docking complex. (**A**) A 3D model and Ramachandran plot assessment of the AmEno15-plasminogen interaction. Each dot on the plot represents the Φ and Ψ angles for each amino acid residue in the docking. For the docking complex molecular dynamics simulation: (**B**) The deformability (green graph) reflects the degree of deformability (0–1 Å). (**C**) The B-factor (pink graph) visualizes flexibility and movement. (**D**) Eigenvalues (purple graph) indicate the energy required for deformation, with lower values showing easier deformation. (**E**) The variance map (cyan bars) identifies complex parts more likely to move during conformational change. (**F**) The covariance map highlights correlated regions between linked amino acids (white: uncorrelated, red: correlated, blue: anti-correlated). (**G**) The elastic network model displays pairs of atoms connected by springs (each dot represents a spring; light grey dots indicate more flexibility).

Four protein–protein dockings were performed using AmEno15 as a receptor, and the proteins spectrin (3LBX), stomatin (7WH3), fibronectin (3M7P), and plasminogen (4DUR) as ligands. For each docking experiment, ten models were generated using ClusPro, with balanced coefficients applied as recommended in the manual because the dominant interaction forces are not known for these complexes. The selection of the best docking model for each pair was based on the lowest energy score: −928.7 kcal/mol for AmEno15-3LBX, −880.6 kcal/mol for AmEno15-7WH3, −1133.4 kcal/mol for AmEno15-3M7P, and −1046.2 kcal/mol for AmEno15-4DUR. All models were further evaluated for quality using Ramachandran plots in PDBsum, with the most favored region percentages being 90.6%, 87.3%, 84.6%, and 76.8%, respectively (Figure 3, Figure 4, Figure 5 and Figure 6, panels A).

Additionally, molecular dynamics simulations were performed using the iMODs server. The green graph of all dockings showed areas of hinges, indicating the degree of deformability measured between 0 and 1 Å. This value was lower than 1 Å in all models. Notably, the docking of AmEno15-7WH3 showed the lowest values of deformability. In contrast, the docking AmEno15-4DUR showed the highest values (Figure 3, Figure 4, Figure 5 and Figure 6, Panels B). The B-factor (pink graphs) indicates protein flexibility and movement. It enables the identification of mobile or static regions within the structure. Regions with high B-factors indicate greater flexibility and movement. Those with low B-factors suggest static regions. Accordingly, our results indicate regions with significant flexibility in the four docking (Figure 3, Figure 4, Figure 5 and Figure 6, Panels C). Regarding the eigenvalues (purple graphs), which represent the energy required to deform the structure, we found the following values for each docking: 6.174995 × 10^−6^ (AmEno15-3LBX), 6.106602 × 10^−5^ (AmEno15-7WH3), 8.125026 × 10^−5^ (AmEno01-3M7P), and 9.918514 × 10^−6^ (AmEno15-4DUR). Since lower values denote easier deformability, these results suggest that docking AmEno15 with spectrin (3LBX) and plasminogen (4DUR) are the most flexible structures. These require less energy to deform (Figure 3, Figure 4, Figure 5 and Figure 6, Panel D). In the conformational change analysis, the variance map highlighted the parts of the docking complex that were more likely to move together. In all docked models, we found high values of cumulative variance (cyan bars), representing significant movements. Low individual variance percentages (purple bars) indicated atoms more likely to remain in place or move together as a rigid unit (Figure 3, Figure 4, Figure 5 and Figure 6, Panels E). The covariance maps represent movement patterns between linked amino acid residues. These patterns result in molecular communication and interactions within the docked structure, revealing key regions of protein function. Our results revealed red regions along the diagonal line of the map in the four docked complexes. These regions represent the binding sites between the receptor and ligand amino acids. In contrast, regions in white and blue outside the diagonal line represent uncorrelated and anti-correlated amino acids, respectively (Figure 3, Figure 4, Figure 5 and Figure 6, Panel F). Lastly, the elastic network model showed that each dot in the graph corresponded to one spring between a pair of atoms. The greyer the dot, the more rigid the spring (grey-black scale). Our results showed dots in light grey along the diagonal, representing a low spring’s stiffness and resulting in highly flexible docked complexes (Figure 3, Figure 4, Figure 5 and Figure 6, Panel G).

### 2.4. Microplate Binding Assays to Stomatin, Spectrin, Plasminogen, and Fibronectin

**Figure 7:**
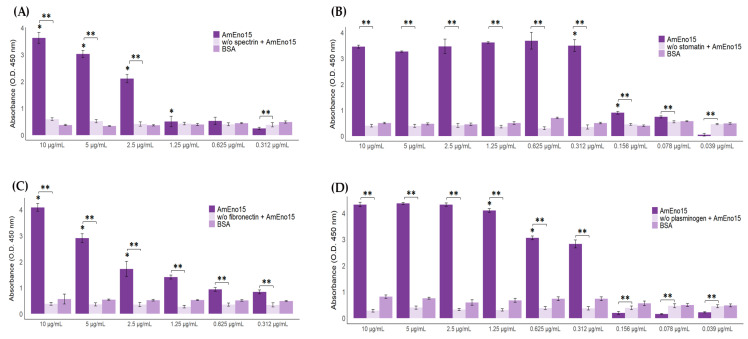
Microplate binding assays of AmEno15 to erythrocyte proteins (spectrin and stomatin), fibronectin, and plasminogen. (**A**) AmEno15-Spectrin binding, (**B**) AmEno15-Stomatin binding, (**C**) AmEno15-Fibronectin binding, and (**D**) AmEno15-Plasminogen binding. Different concentrations of AmEno15 were used in the assay (deep purple). Carbonate buffer-coated wells (only AmEno15) served as specificity controls (light purple). Ligand-coated wells and BSA were used as specificity controls for primary and secondary antibodies, respectively (violet). The assays were performed in three independent experiments, and the absorbance values at O.D. 450 nm are the mean ± standard deviation of triplicate wells. * *p* values < 0.05 and ** *p* < 0.01 were considered statistically significant.

The binding of recombinant AmEno15 to erythrocyte proteins (stomatin and spectrin), fibronectin, and plasminogen was evaluated by a microplate binding assay. We observed that AmEno15 binds to spectrin in a concentration-dependent manner ranging from 10 to 0.312 μg/mL, but not in the BSA control (Figure 7A). Interestingly, the binding assay of AmEno15 to stomatin, with concentrations ranging from 10 to 0.312 μg/mL, showed a similar binding in all cases, but not in a concentration-dependent manner. Nevertheless, when we used lower concentrations (0.312 to 0.039 μg/mL), we did observe binding in a concentration-dependent manner (Figure 7B). As in the previous assay, we did not observe binding to the BSA control. Regarding the AmEno15 to fibronectin binding assay, we observed concentration-dependent binding in all cases (10 to 0.312 μg/mL), and no binding was observed to the BSA control (Figure 7C). Finally, in the assay using plasminogen, we found that at concentrations of 10, 5, and 2.5 μg/mL, there is no significant difference in the binding of AmEno15. Notably, at concentrations 1.25, 0.625, and 0.312 μg/mL, the results suggest a concentration-dependent binding. Nevertheless, in BSA control wells, at concentrations lower than 0.312 μg/mL, we did not observe any binding (Figure 4D). It is essential to note that the control used in all assays (only AmEno15 at different concentrations) yielded absorbances corresponding to non-specific binding, which supports the notion that the binding of AmEno15 depends exclusively on the presence of the ligand.

## 3. Discussion

At present, bovine anaplasmosis is a disease with many unanswered questions and molecular processes that need to be understood. Research has focused on understanding *A. marginale*’s basic mechanisms, such as adhesion and invasion of erythrocytes and adhesion to tick cells [29,30,31]. Ongoing studies aim to identify the key proteins involved and propose new vaccine strategies. In this regard, few studies have addressed the molecules involved in the *A. marginale*–erythrocyte interaction. For example, Noh et al. [29], identified *A. marginale* proteins Msp1a, Mlp3, Am779, and Msp3 as erythrocyte adhesins and Omp13 as a tick cell binding protein. Similarly, the recombinant proteins Msp1a and Msp1b of *A. marginale* expressed in *E. coli* allowed this bacterium to adhere to bovine erythrocytes [31].

Recently, research on *A. marginale* cell adhesion in hosts and vectors is increasing. Thus, we performed immunoinformatics analysis to predict AmEno15’s binding to various ligands. The ligands included erythrocyte membrane proteins, such as spectrin (3LBX) and stomatin (7WH3), as well as proteins linked to pathogenesis, including fibronectin (3M7P9) and plasminogen (4DUR) [9,32,33]. Ramachandran plot scores for the four docked complexes strongly indicate that AmEno15 can interact with all ligands. Molecular dynamics analysis revealed specific amino acid interactions and high deformability. This suggest that AmEno15 may bind ligands involved in several pathogenic processes.

We showed that AmEno15 binds specifically to spectrin and stomatin in a concentration-dependent manner, supporting that *A. marginale* must bind to the membrane for invasion and survival in the host. Therefore, we propose that *A. marginale* uses AmEno15 to bind to stomatin during initial adhesion and that AmEno15 may then bind to spectrin once inside the erythrocyte, as spectrin is found on the inner cytoplasmic face and forms the main structure of the erythrocyte membrane network [34].

In addition, we proved that AmEno15 binds to fibronectin and plasminogen in a concentration-dependent manner. Based on these findings, we hypothesize that *A. marginale* uses AmEno15 as a virulence factor to facilitate invasion of fibronectin-producing cells [35]. This hypothesis is motivated by reports from related species, such as *A. phagocytophilum,* which can infect and lyse endothelial cells—an important step in bacterial infection and invasion [36]. Furthermore, fluorescence microscopy revealed the co-localization of *A. marginale* with an endothelial cell marker in infected calf tissues [30]; therefore, we propose that this fact expands the possibility that *A. marginale* can invade tissue.

We hypothesize two additional roles for AmEno15 in the vector *R. microplus*. First, AmEno15 may participate in the fibrinolysis process by binding to circulating plasminogen in the host’s blood, preventing coagulation and thereby aiding the tick’s blood intake [25]. Second, the AmEno15–fibronectin interaction could play a significant role in *A. marginale* infection of tick gut cells, similar to *Borrelia burgdorferi*, whose membrane proteins bind fibronectin-containing proteins in *Ixodes* spp. tick guts. This interaction may promote the accumulation of bacteria in the tick gut and facilitate migration to the salivary glands [32]. However, these hypotheses require further experimental investigation in *A. marginale*.

After moonlighting proteins were identified in Mexican *A. marginale* strains [10], we prioritized studying AmEno15, since it is considered a virulent strain. Our results show that AmEno15 binds specifically to spectrin, stomatin, fibronectin, and plasminogen; however, its role in these interactions requires further in vivo study. This work offers the first insights into how enolase binds erythrocyte proteins, extracellular matrix components, tick gut proteins, and plasminogen. Our findings contribute to the knowledge of *A. marginale* biology and the elucidation of molecular communication between the vector–pathogen–host triad; however, further studies are required to assess the immunogenicity of AmEno15 or its protective effect on model animals.

## 4. Materials and Methods

### 4.1. Sequence Retrieval

The amino acid sequence of *A. marginale* enolase was obtained from the NCBI database by browsing the genome annotation of strain MEX-15-099-01 (GenBank GCA_008690255.1) and downloading the annotated phosphopyruvate hydratase protein sequence (KAA8472002.1). This sequence, referred to as AmEno15, had its molecular size predicted using ProtParam (https://web.expasy.org/protparam/, accessed on 8 August 2025).

### 4.2. Tridimensional (3D) Modeling

AmEno15 was modeled using SwissModel, a bioinformatics tool that uses protein structure homology to build 3D protein structure models [37]. In this server, models from the AlphaFold DB are appended to the available structures/models. All generated models in SwissModel using AlphaFold DB per-residue scores are transferred to values of pLDDT (predicted local distance difference test) from the underlying template to provide a global model evaluation, where pLDDT is a per-residue measure of local confidence scaled from 0 to 100, with higher scores indicating higher confidence and a more accurate prediction.

### 4.3. Molecular Docking and Molecular Dynamics Simulation

The docking of the modeled AmEno15 with four protein ligands was performed in the ClusPro server to analyze their binding affinity [38]. The proteins used were plasminogen (PDB: 4DUR), fibronectin (PDB: 3M7P), spectrin (PDB: 3LBX), and stomatin (PDB: 7WH3). For each molecular docking, ten models were generated in ClusPro and downloaded in PDB format, then visualized in UCSF ChimeraX [39]. The model with the highest score was selected.

Molecular dynamics simulations were performed on the iMODS server (https://imods.iqf.csic.es//, accessed on 8 August 2025) to evaluate the stability of the docking complex (AmEno15 and each ligand) by assessing deformability, B-factors, covariance maps, variances, elastic network models, and eigenvalues. These parameters assess docked complex motions, which are typically dynamic and involve significant collective conformational changes.

### 4.4. AmEno15 Recombinant Expression

AmEno15 was subcloned into plasmid pET32a(+), and the expression was carried out in *Escherichia coli* strain Rosetta (DE3). This plasmid contains a thioredoxin tag (Trx) and a 6-histidine tag (6His), both of which are in frame with the AmEno15 sequence. Cultures transformed with recombinant plasmids were grown in Luria-Bertani medium (Sigma Aldrich, St. Louis, MO, USA) containing 100 mg/mL ampicillin and induced with 0.8 mM isopropyl thio-β-D-galactoside (IPTG, Sigma Aldrich, St. Louis, MO, USA). Before induction, cultures were grown at 37 °C to an A600 of 0.6–0.8, and the effects of two time points (4 and 16 h) and two temperatures (16 °C and 37 °C) were evaluated. After selecting the induction condition at 37 °C for 4 h, the cells were pelleted by centrifugation at 6000× *g* for 30 min and stored at −80 °C. The cells were lysed by incubation in 50 mM sodium phosphate buffer (10 mL per g wet weight), pH 8.0, containing 300 mM NaCl (Sigma Aldrich, St. Louis, MO, USA), 1 mg/mL lysozyme (Thermo Scientific, Waltham, MA, USA), and 1 mM phenylmethanesulfonyl fluoride (PMSF, Sigma Aldrich, St. Louis, MO, USA) for 30 min on ice and then sonicated for six cycles, each of 15 s with 15 s of cooling between successive bursts at 5 output in a sonifier (Branson 450, Emerson, MX). The lysate was centrifuged at 20000× *g* for 30 min in a Beckman Ultracentrifuge (model LE-80K, 70 Ti rotor). The recombinant AmEno15 was purified from the soluble fraction using Ni-NTA (Thermo Scientific, Waltham, MA, USA) affinity chromatography, which is suitable for His6-tagged proteins.

### 4.5. Microplate Binding Assays to Erythrocyte Membrane Proteins, Plasminogen, and Fibronectin

Microplate assays tested AmEno15 binding with erythrocyte membrane proteins (stomatin, spectrin), plasminogen, and fibronectin. A 96-well ELISA plate (Costar, Fisher Scientific, Waltham, MA, USA) was coated with 100 μL of 3 μg/mL spectrin (S3644, Sigma-Aldrich, St. Louis, MO, USA, Uniprot P02549), stomatin (ab132752, Abcam, Cambridge, MA, USA, Uniprot P27105), plasminogen (SRP6518, Sigma-Aldrich, St. Louis, MO, USA, Uniprot P00747), or fibronectin (F2006, Sigma-Aldrich, St. Louis, MO, USA, Uniprot P02751), and incubated overnight at 4 °C. Plates were washed with 100 μL PBST (0.05% Tween-20) (P4417 and P9416, Sigma Aldrich, St. Louis, MO, USA), then blocked with 100 μL 5% BSA-PBST 0.05% for 2 h at 37 °C. After blocking, plates were washed with 100 μL of 0.05% PBST, and 100 μL of recombinant AmEno15 was added (10 to 0.039 μg/mL), followed by a 2 h incubation at 37 °C. Plates were washed with 100 μL PBST 0.05%. Then, 100 μL Trx tag antibody (mAb, mouse, 1:1000, Cat. A00180-40, GeneScript, Piscataway, NJ, USA) was added and incubated at 37 °C for 1 h. After another PBST wash, 100 μL HRP-conjugated goat anti-mouse IgG (H + L) (1:10,000, Cat. 31430, Thermo Scientific, Waltham, MA, USA) was incubated at 37 °C for 1 h. After washing, 100 μL of 1-Step™ Ultra TMB-ELISA Substrate (Thermo Scientific, Waltham, MA, USA) was added and incubated for 15 min at 37 °C. The reaction was stopped with 100 μL of 2 M sulfuric acid (Baker), and the absorbance was measured at 450 nm using a Multiskan FC (Thermo Scientific, Waltham, MA, USA). Each assay included three independent experiments with three replicates each. For spectrin and fibronectin, AmEno15 concentrations were 10, 5, 2.5, 1.25, 0.625, and 0.312 μg/mL; for stomatin and plasminogen, the concentrations were 1.25, 0.625, 0.312, 0.156, 0.078, and 0.039 μg/mL. Primary and secondary antibody specificity was controlled using ligand-coated wells with BSA (10–0.039 μg/mL, Sigma Aldrich, St. Louis, MO, USA) and carbonate buffer-coated wells with AmEno15 (10–0.039 μg/mL) as specificity controls.

### 4.6. Statistical Analysis

Statistical significance was calculated by one-way ANOVA with Duncan’s Multiple Range Test (DMRT) in STATISTICA version 7.0. *p* values < 0.05 were considered significant. Measurements were performed in triplicate with three repetitions. Plots were made in RStudio 2025.09.0+387.

## Data Availability

Data generated or analyzed during this study are available in the published article.
